# Understanding the Anisotropy in the Electrical Conductivity of CuPt_B_-type Ordered GaInP Thin Films by Combining *In Situ* TEM Biasing and First Principles Calculations

**DOI:** 10.1021/acsaelm.2c00415

**Published:** 2022-07-14

**Authors:** Gemma Martín, Catalina Coll, Lluís López-Conesa, José Manuel Rebled, Enrique Barrigón, Iván García, Ignacio Rey-Stolle, Carlos Algora, Albert Cornet, Sònia Estradé, Francesca Peiró

**Affiliations:** † Laboratory of Electron Nanoscopies (LENS-MIND), Department of Electronics and Biomedical Engineering, Universitat de Barcelona, 08028 Barcelona, Spain; ‡ Institute of Nanoscience and Nanotechnology, Universitat de Barcelona (IN2UB), 08028 Barcelona, Spain; § Scientific and Technological Centers, Universitat de Barcelona (CCiT-UB), 08028 Barcelona, Spain; ∥ Instituto de Energía Solar, Universidad Politécnica de Madrid, Avda. Complutense 30, 28040 Madrid, Spain

**Keywords:** CuPtB ordering, GaInP thin film, DFT simulation, APDB, TEM biasing

## Abstract

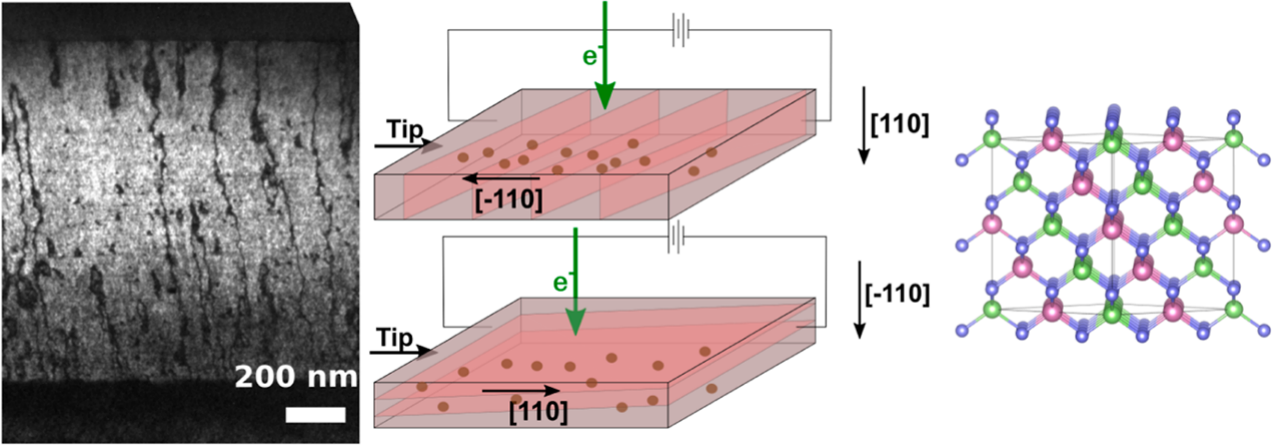

In this work, the effect of CuPt_B_ ordering on the optoelectronic properties of Ga_0.5_In_0.5_P is studied by combining *in situ* transmission electron microscopy measurements and density functional theory (DFT) calculations. GaInP layers were grown by metal organic vapor phase epitaxy with a CuPt_B_ single-variant-induced ordering due to the intentional misorientation of the Ge(001) substrate. Moreover, the degree of order was controlled using Sb as the surfactant without changing other growth parameters. The presence of antiphase ordered domain boundaries (APDBs) between the ordered domains is studied as a function of the order parameter. The *in situ* electrical measurements on a set of samples with controlled degree of order evidence a clear anisotropic electrical conductivity at the nanoscale between the [110] and [1–10] orientations, which is discussed in terms of the presence of APDBs as a function of the degree of order. Additionally, DFT calculations allow to determine the differences in the optoelectronic properties of the compound with and without ordering through the determination of the dielectric function. Finally, the anisotropy of the electrical conductivity for the ordered case is also discussed in terms of the effective mass calculated from the band structure on specific *k*-paths. By comparing the experimental measurements and the theoretical calculations, two factors have been presented as the main contributors of the electric conductivity anisotropy of CuPt_B_-type ordered GaInP thin films: antiphase boundaries that separate domains with uniform order (APDBs) and the anisotropy of the effective mass due to the alternating of In/Ga rich planes.

## Introduction

III–V semiconductors have been explored as active materials for high-speed electronic devices, many types of optoelectronic devices, and high-efficiency photovoltaic devices. The widespread use of III–V semiconductors, such as GaInP, is due to the inherent advantages of direct band gap and high electron mobility.^1^ Indeed, GaInP is a key material in III–V MultiJunction solar cells, which are the most efficient solar cells till date achieving conversion efficiencies in excess of 35% for space applications^2^ and reaching 47.1% in high concentrator setups.^3^

GaInP is prone to present a CuPt ordering in the group III sublattice as many other III–V ternaries. This ordering consists of alternating Ga- and In-rich {111} planes on the zinc-blende structure. For (001)-oriented substrates, this ordering affects the (−111) and (1–11) planes, that is, a CuPt_B_ type with two variants that can be selected by substrate misorientation. In particular, a misorientation of the (001) substrate by a few degrees toward (111) favors the formation of a single variant.^4^ In the past, it was also demonstrated that the substrate misorientation angle plays a determinant role to define the degree of order and the extension angle of the antiphase ordered domain boundaries (APDBs) separating domains of uniform ordering, as shown for Ga_0.48_In_0.52_P layers grown on InP^5^ or for Ga_0.48_In_0.52_P layers grown on GaAs substrates.^6^

Besides substrate misorientation, surfactants, such as Sb among others,^7^ can be used to control the degree of order, described as Ga_0.5(1−η)_In_0.5(1+η)_P/Ga_0.5(1+η)_In_0.5(1−η)_P, where η is defined as the order parameter (η = 0 for a fully disordered GaInP material and η = 1 for a completely ordered material) without changing other growth parameters.

Ordering has a direct effect on the electronic structure and therefore on the band gap energy (*E*_g_) of the alloy, and a preferent orientation of ordered domain boundaries in single-variant CuPt-type ordered materials leads to anisotropy in the minority carrier diffusion length and reduces carrier mobility.^8,9^ Therefore, to further increase the performance of the aforementioned devices, a deep understanding of the relationship between the structural and the electrical properties of the devices is mandatory.^10^ From decades ago, electronic structure modifications induced by this ordering have been studied using density functional theory (DFT), such as band gap reduction and band splitting,^11^ strain deformation and effective mass,^12^ or conduction-valence band deformation.^13^ Nowadays, it is worth revisiting these topics as the availability of higher computational power and improved pseudopotentials enable building larger and more complex models to make the most of DFT calculations. In particular, perturbation theory,^14,15^ known since the 1980s to obtain the effective mass considering eight bands, was not used due to the limited computational power at that time, but now it is implemented in ab initio codes, allowing hundreds of bands to be taken into account.^16,17^ Recently, purely *ab initio* calculations of the effect of doping on III–V compounds^18^ have been reported, as well as experimental–theoretical studies where the strain due to the order is calculated and compared with experimental measurements.

The present work focuses on the understanding of the electronic configuration of CuPt_B_-type ordered GaInP thin films grown by metal organic vapor phase epitaxy (MOVPE) on Ge misoriented substrates using Sb as the surfactant to control the degree of order. The electrical conductivity of the GaInP layer is measured at the nanoscale by *in situ* biasing transmission electron microscopy (TEM), and the dissimilar values of carrier mobility observed between [110] and [1–10] directions are discussed in light of ab initio effective mass calculations for ordered and disordered GaInP models and the effect of APDBs.

## Experimental Details

Ga_0.5_In_0.5_P semiconductor structures were grown in a commercial horizontal MOVPE reactor (Aixtron AIX200/4) equipped with an *in situ* reflectance anisotropy spectrometer (Laytec EpiRAS 200) on Ge(001) substrates with 6° misorientation toward the nearest [111]. The precursors used were PH_3_, AsH_3_, TMGa, TMIn, DMZn, and also TESb since Sb was used as a surfactant. The samples consist of a 365 nm thick GaInP nucleation layer, nominally undoped, followed by a Zn-doped, 1100 nm thick GaInP target layer and a 400 nm thick Ga(In)As capping layer. Three samples were grown, each with a different Sb flow during the growth of the GaInP target layer to promote surfactant-mediated disordering. The Sb/P flow ratios used were 0, 411, 728, and 1720 ppm, respectively. All layers were grown at 675 °C, with a V/III ratio of 120 and a growth rate of 0.60 nm/s. Once grown, the dopant concentration on the GaInP target layer was measured by electrochemical capacitance–voltage (ECV) profiling. Further details on the growth of these samples can be found elsewhere.^19,20^

Thin TEM lamellas were prepared by the focused ion beam (FIB, CrossBeam 1560XB, Zeiss, operated at 30 kV) using the lift-out technique.^21^ Two orthogonal cross-sections were obtained from each sample, as shown in the FIB-SEM image acquired during the preparation (see Figure S1) in order to examine the sample along [110] and [1–10] zone axes. TEM characterization and selected area electron diffraction (SAED) observations were performed in a JEOL 2100 TEM operated at 200 kV. *In situ* experiments were performed with the same microscope equipped with a scanning tunneling microscope (STM) holder from Nanofactory used as an *in situ* TEM electrical probe (Figure S2a).

DFT simulations were carried out using ordered and disordered GaInP supercells. Structural relaxation was performed on both models using the Vienna Ab initio Simulation Package (VASP).^22,23^ A cutoff energy of 200 eV for the plane waves was used. A 11 × 11 × 11 Monkhorst–Pack k-point grid in the Brillouin zone was used. For proper band gap calculation, the generalized gradient approximation (GGA)^24^ including the Hubbard model which considers an extra onsite Coulombic interaction^25^ was used, considering the Coulombic energy (U) of −18 eV for all the group III atoms for the disordered structure. Modified Becke–Johnson (mBJ) exchange potential^26^ was considered for the high symmetry ordered case. The relaxed structure was used to compute the energy loss function (ELF) and the complex dielectric function (CDF) by OPTIC^27−29^ task of WIEN2k package.^30,31^ The Perdew–Burke–Ernzerhof (PBE)^32^ functional was used setting RKmax to 7.0 and defining 500 k-points on the Brillouin zone. The energy and charge convergence criterion were set to 0.0001 Ry and 0.001 e, respectively. Spin–orbit splitting was considered to obtain the mass invers tensor by using the mstar code developed for WIEN2k^16^ based on perturbation theory. KVEC from Bilbao Crystallographic Server^33,34^ was used to get the proper coordinates of the high symmetry K-points and SeeK-path to visualize the Brillouin zone of our structures.^35^

## Results

The order parameter (η) of the GaInP samples grown using different Sb/P ratios was determined from the band gap energy, as reported in previous studies.^36^ Photoluminescence (PL) measurements showed a band gap energy reduction as the order parameter increased, as shown in Figure S3. These η and *E*_g_ values as a function of the Sb/P flux (at 20 K) are summarized in Table 1.

**Table 1:** Degree of Order (η) and Band Gap Energy (*E*_g_) at 20 K as a Function of the Sb/P Ratio Used during the Growth

Sb/P (ppm)	η	*E*_g_ (eV)
0	0.53	1.855
411	0.48	1.880
728	0.43	1.904
1720	0.31	1.949

**Figure 1:**
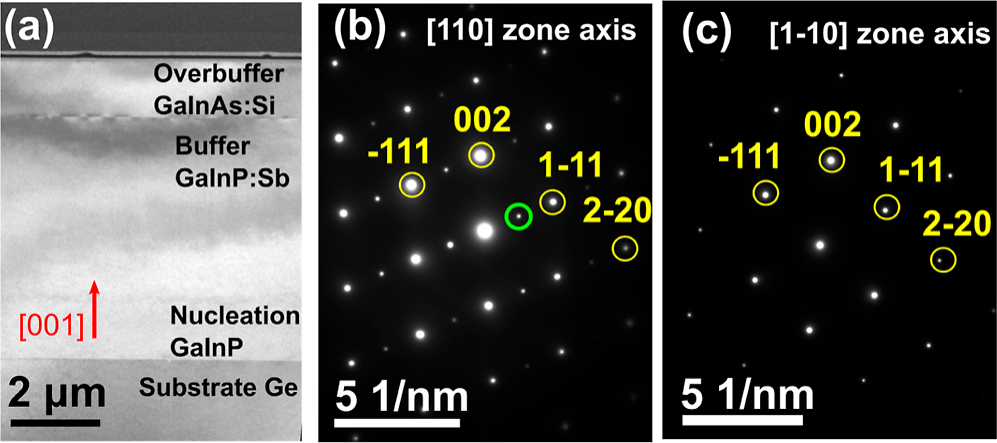
(a) Low-magnification image of the GaInP thin film (No Sb flux) with the layers labeled (highlighted in red, there is the growth direction [001]). (b) Indexed SAED of the lamella prepared in the [110] zone axis (the satellite spots are highlighted in green) and (c) on the orthogonal [1–10] zone axis.

Figure 1a shows the low-magnification TEM image of the Ga_0.5_In_0.5_P semiconductor structures. The presence of a single variant of CuPt_B_-type ordering was also verified by TEM characterization.^20^ The CuPt_B_ order was visible through the presence of superstructure spots in the diffraction pattern only in [110] zone axis orientation (Figure 1b). A correlation between the intensity of the extra spots and the order parameter determined by PL was also observed. No additional spots were observed in any of the [1–10] zone axis views (Figure 1c), confirming an anisotropic ordered structure with alternating In-rich, Ga-rich planes only in (1–11) planes.

**Figure 2:**
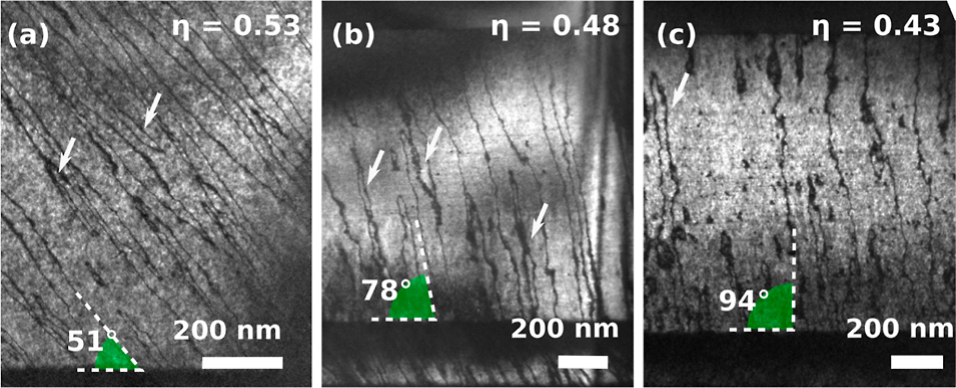
Two-beam DF images of the GaInP:Sb layer with a Sb/P flux of 0 (a), 411 (b), and 728 (c) ppm. The bright contrast corresponds to the ordered domain and the darker to the APDBs between them. In green, the extension angle (the angle of APDBs with respect to the plane of the interface) of APDBs is highlighted. The small arrows point the closed loops.

Two-beams dark field (DF) images were acquired using the extra *g* = (1/2, −1/2, and 1/2) spot, so the bright contrast corresponds to the regions with order. The dark narrow regions can be identified as APDBs. Most of these APDBs present quasi parallel traces when viewed along the [110] (Figure 2). Very few APDB closed loops defining a thin domain [110] projection are also visible (see the small arrows). Although we cannot disregard the presence of some APDB closed loops in the perpendicular [1–10] orientation,^37^ we were not able to reveal them with enough contrast neither by DF nor by high-angle annular dark field (HAADF) high-resolution imaging.^20^ Thus, these DF two-beam images prove the presence of APDBs between the ordered domains (Figure 2a–c). For the sample grown using 1720 ppm (order parameter 0.31), the low intensity of the satellite *g* = (1/2, −1/2, and 1/2) impeded the acquisition of DF images with significant contrast. The orientation, extension, and width of the domains were quantified in order to detect any possible existing relationship with the degree of order. In addition, the linear density of APDBs was calculated considering it as the number of APDBs divided by the total length of a line profile, as illustrated in Figure S4a.

Additional geometrical measurements related to the APDB distribution are summarized in Table 2 {domains width (nm), ordered area fraction (%), extension angle (°) [considered as the angle of APDBs measured counterclockwise from the plane of the interface toward the ordered (1–11) planes]}. The error was estimated as the standard deviation. The ordered fraction was computed as the ratio of dark/bright areas after defining an intensity threshold to segment the image. Since these thresholds depend on the two-beam imaging conditions, that can slightly differ between different samples, this ratio must be just considered as a qualitative indicator.

**Table 2:** Quantitative Study of the Ordered Domains and the Antiphase Domain Boundaries (APDBs): Domain Width (nm), Ordered Area Fraction (%), Extension Angle (°) (the Angle of APDBs with Respect to the Plane of the Interface), and Linear Density of APDBs (nm^–1^ ‰) Measured for Different Sb/P Fluxes and Degree of Order (η)

Sb/P (ppm)|η	0|0.53	411|0.48	728|0.43
width (nm)	52	61	172
area ordered (%)	85	81	74
angle (deg)	51	78	94
linear density APDBs (nm^–1^ ‰)	1.6	1.3	1.2

By plotting the dependence of the four parameters as a function of the flux (Figure S4b), it can be observed that the extension angle and the linear density are the only variables with a clear dependence on the flux/order parameter. Thus, certain spatial asymmetry is introduced in the GaInP:Sb layer due to the presence of the CuPt-type B ordering and the APDBs. Moreover, the spatial asymmetry presents a dependence with the degree of order.

Therefore, as can be seen in Table 2 and Figure S4, as the Sb/P flux increases (or in other words, as the degree of order η decreases), the domains width increases, and the linear density of APDBs decreases.

**Figure 3:**
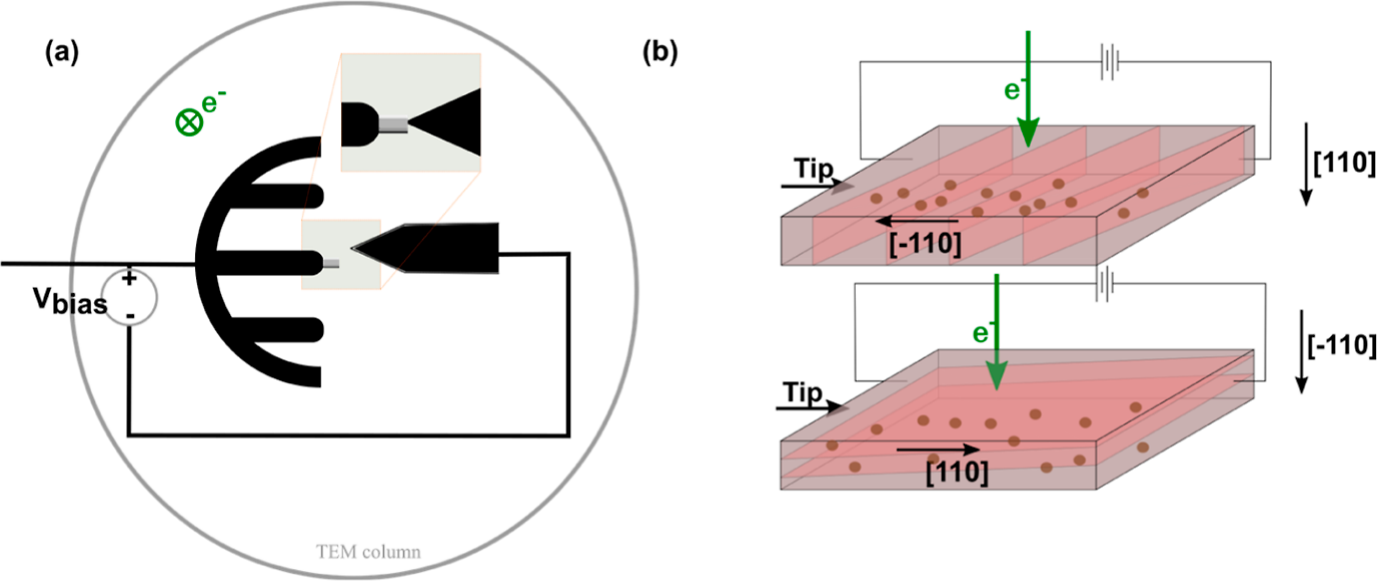
(a) Schematic representation of the *in situ* TEM-STM system. A sharp Pt tip is attached to the movable part of the STM holder, and both the samples and the STM tip are oriented perpendicular to the electron beam. (b) Scheme of the lamella contact: (top) the lamella is oriented on the [110] direction, and the current flow is measured along [−110], and (bottom) the lamella is oriented on the [−110] direction, and the current flow is measured along [110]. APDBs are represented as red planes in the layer. The current flow is schematized by the brown dots.

To explore the effects of this single-variant CuPt_B_ ordering and APDBs on the electrical properties of the material, *in situ* biasing TEM electrical measurements were performed to analyze the difference on conductivity along specific directions at the nanoscale. For these measurements, a new set of samples was prepared by FIB.^38−41^Figure 3 shows the schematic of the TEM-STM holder, where a sharp platinum tip was attached to the movable part of the STM holder, and both the GaInP samples and the STM tip were oriented perpendicular to the electron beam.^42,43^ As can be seen in the figure, the protective Pt layer grown during FIB preparation was also etched by FIB in order to cut the Pt metallic path forcing the current to flow through the GaInP layer (see Figure S2b). During the measurements, the movable tip of the TEM-STM holder was positioned to contact directly on the GaInP:Sb layer to perform the electrical measurements solely through the GaInP layer.

The local conductivity of both [110] and [1–10] oriented samples was measured for each Sb/P ratio by *in situ* biasing inside the TEM. A ramp from 1 to −1 V, with 400 sampling points and an acquisition time of 100 ms for the entire ramp was used to calculate the electrical resistivity (ρ) of each GaInP:Sb layer in both directions. Three *I*–*V* curve measurements were acquired for each sample, moving slightly the position of the tip for each measurement in order to minimize the effect of variations in the quality of the tip/sample contact in the resistance calculations. The influence of the tip/semiconductor rectifying contact can be eliminated by performing the fit at higher voltages (as shown in Figure S5) where the *I*–*V* curve is vastly dominated by the resistive behavior of the GaInP:Sb layer.^44^

The degree of anisotropy in the electrical conductivity was calculated as the ratio between the mobility in the [110] oriented sample and the mobility of the [1–10] oriented sample, as is schematized in Figure 3b. The mobility (μ) in each crystal direction and its ratio were calculated from resistivity (ρ) values and dopant concentration (*N*_A_). The resistivity (ρ) was calculated from the resistance obtained from the ohmic region of the *I*–*V* curves (see Figure S5) and the geometric parameters of the GaInP structures obtained from the TEM images and considering the thickness of the TEM lamella to be 100 nm (see Table 3). NA was measured by ECV profiling. In all linear regressions obtained for the resistance calculations, the *r*^2^ was larger than 0.9. It is worth to mention now that the current flows through the [1–10] direction in the case of the [110] oriented lamella preparation and along [110] direction in the [1–10] oriented sample (Figure S1). For this reason, from now on we will name these ρ values as ρ_[1–10]_ and ρ_[110]_.

**Table 3:** *In Situ* Resistivity Measurements in Both Crystal Directions Compared with the Degree of Order, the Doping of the Layers, and the Sb/P Ratio^a^

Sb/P (ppm)	η (%)	*N*_A_ (cm^–3^)	ρ_[1–10]_ (Ω·m)	μ_[1–10]_ [cm^2^/V s]	ρ_[110]_ (Ω·m)	μ_[110]_ [cm^2^/V s]	μ_[110]_/μ_[1–10]_
0	53	8.9 × 10^16^ ± 2.2 × 10^16^	63.0 ± 4.0	111 ± 35	11.5 ± 0.5	611 ± 179	5.5
411	48	1.5 × 10^17^ ± 3.8 × 10^16^	43.9 ± 0.2	95 ± 24	10.6 ± 0.1	393 ± 102	4.1
1721	31	4.3 × 10^17^ ± 1.1 × 10^17^	5.46 ± 0.02	266 ± 68	4.95 ± 0.01	294 ± 74	1.1

a The mobility in each crystal direction and its ratio have been also calculated from resistivity values and dopant concentrations.

Table 3 shows that as the Sb/P ratio increases (or in other words, as the order parameter decreases) the resistivity in both [1–10] and [110] directions diminishes. To some extent, this is expected as increased Sb concentrations during the MOVPE growth boost the incorporation of Zn into the solid,^45^ hence yielding a higher dopant concentration in the GaInP:Sb layer. However, superimposed to this increase in free carrier concentration, there is an evident change in the hole mobility with a notable asymmetry between both directions. Mobilities in the [1–10] direction are significantly lower than that in the [110] direction, being this difference larger with growing order in the alloy. In other words, ordered GaInP samples show very different mobilities between [1–10] and [110] directions, whereas in disordered samples, such mobilities tend to converge to similar values. Thus, *in situ* TEM measurements of the local conductivity of GaInP structures have demonstrated the correlation between the ordering degree (modulated by the Sb/P ratio used in the MOVPE growth) and the anisotropic mobilities in [1–10] and [110] directions. As can be seen in Table 3, as the degree of order decreases, the degree of anisotropy calculated as the ratio μ_[110]_/μ_[1–10]_ decreases too. Moreover, comparing these results with the distribution and density of ordered domain boundaries previously measured (see Table 2), it can be seen that the degree of anisotropy is reduced as the linear density of the APDBs decreases and the domain width increases. This result reveals that APDBs contribute significantly to the electrical conductivity anisotropy.

The APDBs, as unveiled using two-beams DF TEM imaging, present an extension angle increasing when increasing the Sb/P ratio, yielding values even higher than 90°. The correlation of the electrical measurements at the nanoscale with these results shows that the resistivity increases with the order parameter when the current flows across the APDBs (*i.e.* ρ_[1–10]_), and it is kept relatively independent from the order parameter when the current flows almost tangent to the APDBs (*i.e.* ρ_[110]_). These results could be explained, at least in part, by the fact that APDBs are two-dimensional defects in the crystalline structure whose asymmetric distribution induces asymmetric resistivity values^46^ because of their role as active recombination centers.^47−51^ Additional effects should be also mentioned to have a more complete vision. First, the band alignment and corresponding offset variation between ordered and disordered regions exhibiting different band gap depending on the degree of order^52^ can induce local potential barriers that can also affect the conductivity. Second, the existence of sections of the APDB closed loops oriented almost perpendicular to the (1–10 plane) (then, the electrical current flowing across these segments when measuring ρ_[110]_) may reduce the global anisotropy related to the asymmetric distribution of APDB in the single-variant ordering.

Besides these APDB-related effects, a deeper study of the effect of the ordering on the optoelectronic properties should be made to discuss other possible contributions to the conductivity anisotropy.^12,53^ First principles calculations of idealized GaInP with and without order are the best model systems as they allow neglecting the effect of the APDBs.

Two structures were built to model ordered and disordered GaInP films, by removing the symmetry of an initial 2 × 2 × 2 GaP cell.^54^ The group III positions on the (1–11) planes were randomly occupied by In/Ga to get a disordered GaInP or alternatively by In or Ga to get a single-variant CuPt_B_-ordered GaInP. The cell parameter was fixed to be double of the one found experimentally, 11.32 Å. Thereon two models P1-symmetry 64 non-equivalent atom structures were used as the input for DFT calculations (Supporting Information, Figure S6). Full structural relaxation using the GGA method was applied to both structures by force and energy minimization. The optimized structures lose their cubic shape (Supporting Information, Table S1), and there is a small change in the atomic positions.

DFT calculations usually underestimate the value of the band gap energy. To obtain a more reliable band gap value, hybrid potentials can be used such as GGA + *U* or mBJ. Here, the GGA + *U* method was used, the value of effective energy (*U*) applied to In and Ga d orbitals was adjusted until the band gap energy reached a value similar to the experimental one,^55^ and the optimum value was set to −18 eV. The band gap calculations using DFT (as summarized in Table 4) yield a narrower band gap for the fully ordered model. Even if the calculated *E*_g_ values cannot be strictly correlated with the experimental results from the real samples (the simulations consider fully disordered and ordered models and any possible effect of the substrate is neglected), ^19^they exhibit the same tendency with the degree of order as the experimental *E*_g_ values measured by PL (see Table 1), giving also a band gap narrowing as the degree of order increases. This tendency also agrees with the results reported by other experimental^55^ and theoretical studies,^52^ although the absolute values differ because of the different hybrid potentials used in the respective DFT calculations.

**Table 4:** Summary of the Band Gap Energy (*E*_g_), Electron Effective Mass (*m*_e_*), Heavy/Light Holes Effective Mass (*m*_hh_*/*m*_lh_*), Energy Splitting of the Valence Band [Heavy–Light Holes (*E*_hh_–*E*_lh_), and Heavy Hole–Split Orbit (*E*_hh_–E_so_)] Computed for Both Structures

	ordered	disordered
*E*_g_	1.785	2.188
*m*_e_*	0.092 m_0_	0.090 m_0_
*m*_hh_*	0.234 m_0_	0.198 m_0_
*m*_lh_*	0.185 m_0_	0.189 m_0_
*E*_hh_–*E*_lh_	57 meV	17 meV
*E*_hh_–*E*_so_	285 meV	107 meV

**Figure 4:**
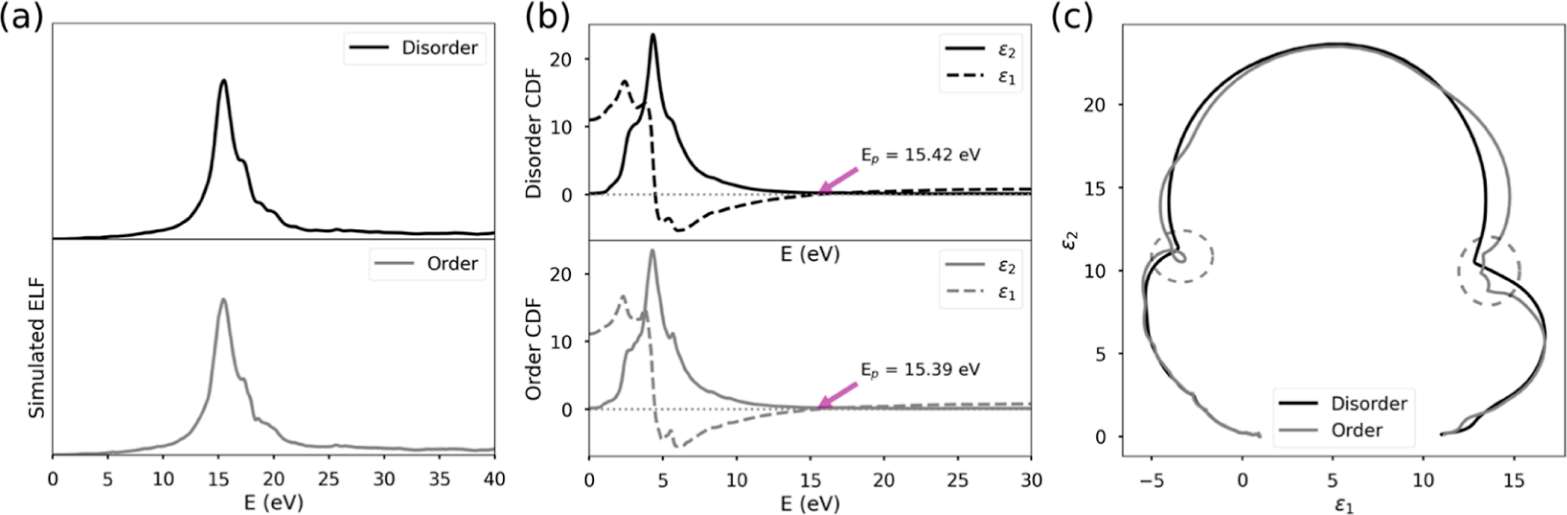
(a) ELF from the DFT simulation (black: disorder and gray: order). (b) CDF of the simulated structures (top-black: disorder and bottom-gray: order). (c) Cole–Cole diagram from the calculated CDF (black: disorder—gray order) dashed circles highlight the inter-band transition fingerprint.

The effect of the degree of order on the optical properties of the GaInP was further examined from the calculated ELF and CDF. Figure 4a displays the ELF obtained from the DFT calculations for both models. For both structures, the real part of the CDF (Figure 4b) crosses the energy axis with the positive slope at 15.5 eV, in good agreement with the experimental bulk plasmon energy of the samples.

Moreover, the simulations clearly establish that extra inter-band transitions are allowed for the ordered case as the ELF post-peak region shows a finer structure. The same conclusion is reached by comparing both Cole–Cole diagrams from where it can be determined that extra inter-band transitions are allowed for the ordered case (Figure 4c). The inter-band transitions are also visible in the imaginary part of the CDF (ε_2_), where they correspond to the main peaks (Figure 4b). The plots show that for the ordered structure, there are three sharp peaks, while for the disordered structure, only one peak is clearly visible with a shoulder at each side. The density of states (DOS), alongside with the CDF, allows the determination of the nature of these transitions. As shown in Supporting Information (Figure S7), for the disordered model, three main inter-band transitions are observed in the ELF and identified in the CDF at 17, 18.6, and 19.8 eV; they correspond to a transition between the d occupied band to unoccupied p (d → p) for In and Ga atoms. On the other hand, the ordered model presents an extra transition at 21.5 eV, clearly visible in the ε_2_; this transition is a d → p transition for In atoms.

Interestingly, DFT also enables for the calculation of the effective mass inverse tensor. The effective mass tensor calculation was performed using perturbation theory for both structures considering 1k bands. The electron effective mass for the conduction band was found to be 0.092 m_0_ for the ordered case and 0.090 m_0_ for the disordered one. Also, the effective mass for the heavy and light holes was computed for the valence band, which would be responsible of the conductivity in our experimental samples. The results (see Table 4) are also in good agreement with the work performed by Emanuelsson *et al.* using perturbation theory on an eight bands model.^56,57^

A high symmetry structure was extracted from the relaxed ordered GaInP where the new structure has *R*3*m* symmetry, space group 160. The *R*3*m* structure presents four non-equivalent atomic positions occupied by Ga, In, and two P atoms, the lattice parameters were found to be *a* = *b* = *c* = 6.96 Å, and the angles α = β = γ = 33.41° (Figure S8). The *R*3*m* structure allows the calculation of the band structure for the ordered GaInP. mBJ exchange potential was used in addition to the GGA potential to improve the band gap accuracy.

**Figure 5:**
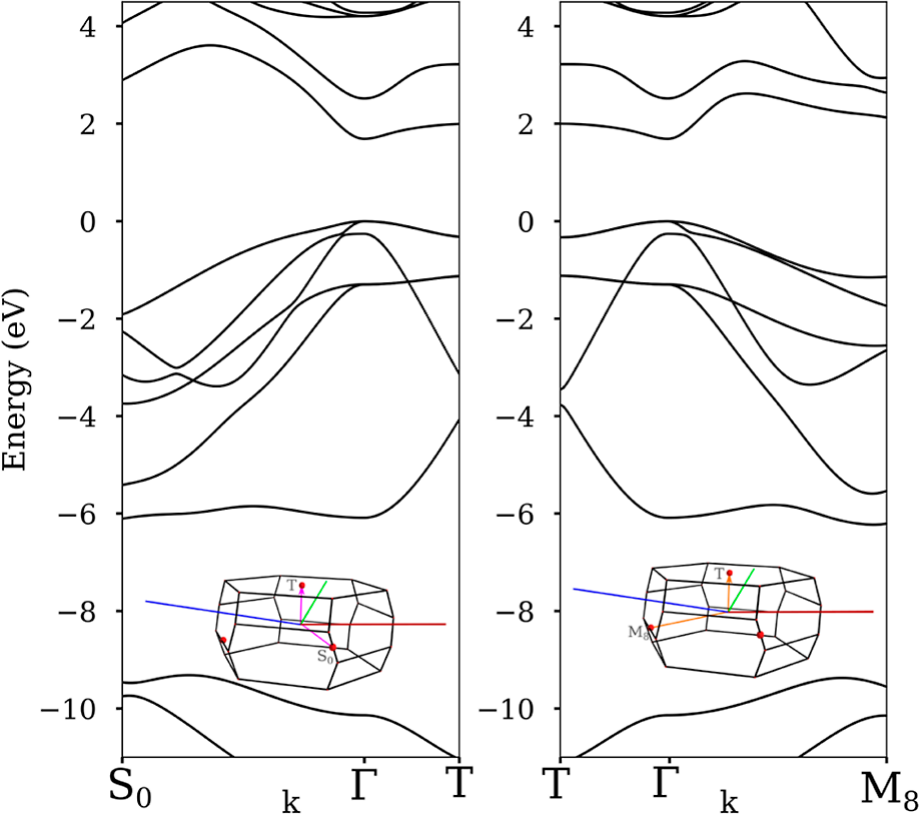
Band structure for GaInP *R*3*m* computed along the *k*-path S_0_–Γ–T (right) and T−Γ–M_8_, the *k*-path is displayed on the Brillouin zone plotted in the inset.

The anisotropic behavior of the effective mass is illustrated by plotting the band structure along a *k*-path: S_0_–Γ–T and T−Γ–M_8_. This specific k path was designed by finding the *k* points correlated with the planes with the alternation of In/Ga (−1–1–1) [represented by T: *k* = (−0.5, −0.5, −0.5)] and its perpendiculars (−1–12) and (1–10) [represented by M_8_: *k* = (−0.34, −0.34, 0.18) and S_0_: *k* = (0.34, −0.34, 0.0), respectively], parallel to the planes of the *R*3*m* crystalline structure. As can be seen in Figure 5, the band structure is asymmetric with respect to the Γ point along both specific *k*-paths, in agreement with previously reported studies.^12,13^ The maximum of the valence band has been fitted to a parabolic function obtaining a different fit by each side of the maximum. The theoretical effective mass ratio has been calculated for the fully ordered case, obtaining a ratio of 1.5. In addition, if the anisotropy is quantified according to the expression , where *m*_∥_ is the effective mass in the ordering direction and *m*_⊥_ on the perpendicular one, in agreement of the value reported for the fully ordered case by Franceschetti *et al*.^12^ From these results, we can conclude that not only APDBs but also the anisotropic behavior of the effective mass has a role in the anisotropy of the electrical conductivity of CuPt_B_-type ordered GaInP thin films observed by *in situ* TEM.

## Conclusions

The effect of CuPt_B_ ordering on the electronic properties of GaInP has been assessed by *in situ* TEM experiments and DFT simulations. With *in situ* TEM, the anisotropy of the conductivity between [110] and [1–10] orthogonal directions has been determined from the electric measurements through the GaInP:Sb layer for two orthogonal oriented samples. It has been also observed that as the degree of order decreases, the degree of anisotropy of the electrical conductivity decreases too. Moreover, ordered GaInP samples have shown very different hole mobilities between [1–10] and [110] directions, whereas in disordered samples, such mobilities tend to converge to similar values. Furthermore, the resistivity increases with the order parameter when the current flows across the APDBs (*i.e.* ρ_[1–10]_), it is kept relatively independent from the order parameter when the current flows tangent to the APDBs (*i.e.* ρ_[110]_), and the degree of anisotropy is reduced as the linear density of the APDBs decreases. This result reveals that APDBs are an important contributor to the electrical conductivity anisotropy. From DFT simulations, the band structure is clearly affected by the ordering. A decrease in the energy band gap occurs as the order degree increases, as shown by PL; extra inter-band transitions are allowed; in addition, the splitting of the orbitals of the valence band increases. All three factors are crucial for the determination of the optical properties of the compound, but they do not explain the anisotropy of the conductivity. To determine the atomic alternation effect in the anisotropy, the band structure for a wise *k*-path for the fully ordered structure has been computed showing that the effective mass presents an anisotropy on the specific direction due to the ordering. To conclude, the results obtained by *in situ* TEM and DFT calculations show that not only the anisotropy of the conductivity can be attributed to the presence of APDBs, whose extension angle and density are dependent on the Sb/P ratio used during the growth, but also that the anisotropic behavior of the effective mass can affect the anisotropy of the electrical conductivity of CuPt_B_-type ordered GaInP thin films.

## Supporting Information Available

SEM image of the sample during the TEM lamella preparation; in situ holder used and TEM images of the experimental setup; lattice parameters of ordered and disordered structure before and after the structural optimization; room-temperature PL spectra of GaInP:Sb layers grown with different Sb/P ratios; scheme of the layer stack and APDB appearance; measured parameters as a function of the degree of order /flux of Sb; example of the I–V characteristics for M–S–M structures; structures before relaxation of ordered GaInP and disordered GaInP; real part and imaginary part of the CDF computed for the disordered and ordered structures; and crystallographic information of GaInP R3m (PDF)
