# Supplementary material for: Understanding the Anisotropy in the Electrical Conductivity of CuPtB-type Ordered GaInP Thin Films by Combining In Situ TEM Biasing and First Principles Calculations
Source: ACS Appl Electron Mater. 2022 Jul 14;4(7):3478–85. doi: 10.1021/acsaelm.2c00415 (PMC9344399; doi:10.1021/acsaelm.2c00415)
Supplement: Supplementary file 1 — el2c00415_si_001.pdf [file el2c00415_si_001.pdf]

Supporting Information

Understanding the anisotropy in the electrical conductivity of $CuPt_B$ type ordered GaInP thin films by combining *in-situ* TEM biasing and first principles calculations.

Gemma Martín[1,2,3 ‡], Catalina Coll[1,2 ‡ *], Lluís López-Conesa[1,2,3], José Manuel Rebled[1,2,3], Enrique Barrigón[4], Iván García[4], Ignacio Rey-Stolle[4], Carlos Algora[4], Albert Cornet[1], Sònia Estradé[1,2], Francesca Peiró[1,2]*

*Corresponding authors: gemmamartin@ub,edu, ccollbenejam@ub.edu

‡Equal contribution

[1]Laboratory of Electron Nanoscopies (LENS-MIND), Departament of Electronics and Biomedical Engineering, Universitat de Barcelona, 08028, Barcelona, Spain

[2]Institute of Nanoscience and Nanotechnology, Universitat de Barcelona (IN2UB), 08028 Barcelona, Spain

[3]Scientific and Technological Centers, Universitat de Barcelona (CCiT-UB), 08028 Barcelona, Spain

[4]Instituto de Energía Solar, Universidad Politécnica de Madrid, Avda. Complutense 30, 28040 Madrid, Spain

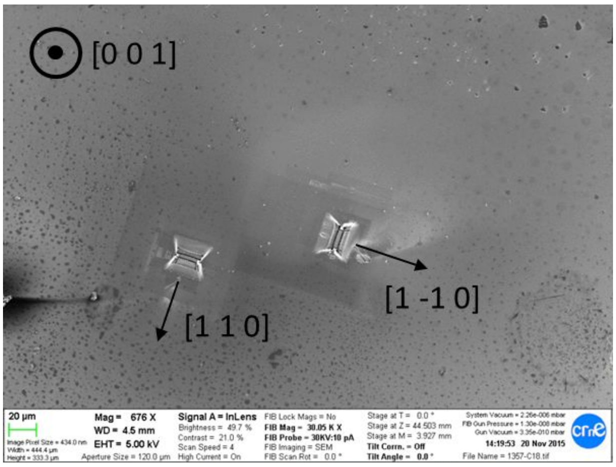

Fig. S1 SEM image of thesample during the TEM lamella preparation in the two orthogonal [110] and [1-10] cross-section TEM orientations.

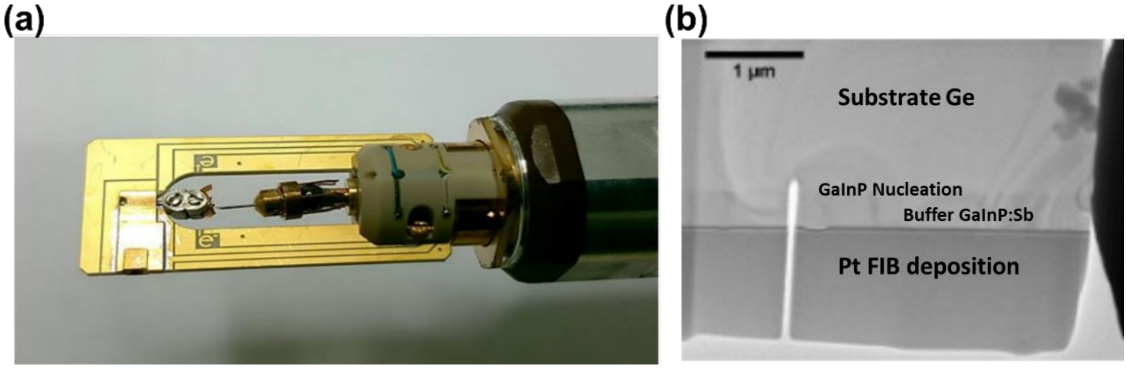

Fig. S2 (a) *In-situ* holder used in this work with the Pt tip on the right-hand side and a TEM grid attached to a Pt wire as a sample support on the left-hand side. A sapphire ball is supported by a piezotube to drive the hat, the only moving part in the holder where the STM tip is placed. The probe can be three-dimensionally positioned enabling the selection of a specific nanostructure, contacting it and applying a voltage to perform electrical measurements. (b) TEM image of the experimental setup

	a (Å)	B (Å)	C (Å)	α (°)	β (°)	γ (°)	Eg (eV)
**Disordered**	11.31920	11.31920	11.31920	90.000	90.000	90.000	
Relaxed	11.25265	11.26736	11.27037	89.9988	90.0178	90.0176	2.188
**Ordered**	11.31920	11.31920	11.31920	90.000	90.000	90.000	
Relaxed	11.27918	11.27918	11.27918	89.8153	89.8153	89.8153	1.785

Table S1 Lattice parameters of ordered and disordered structure before and after the structural optimization.

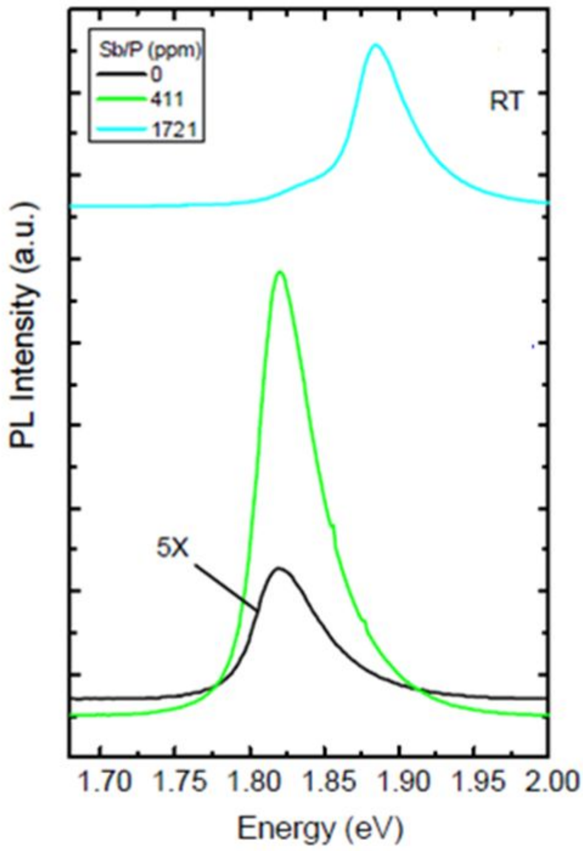

Fig. S3 Room temperature PL spectra of GaInP:Sb layers grown with different Sb/P ratios.

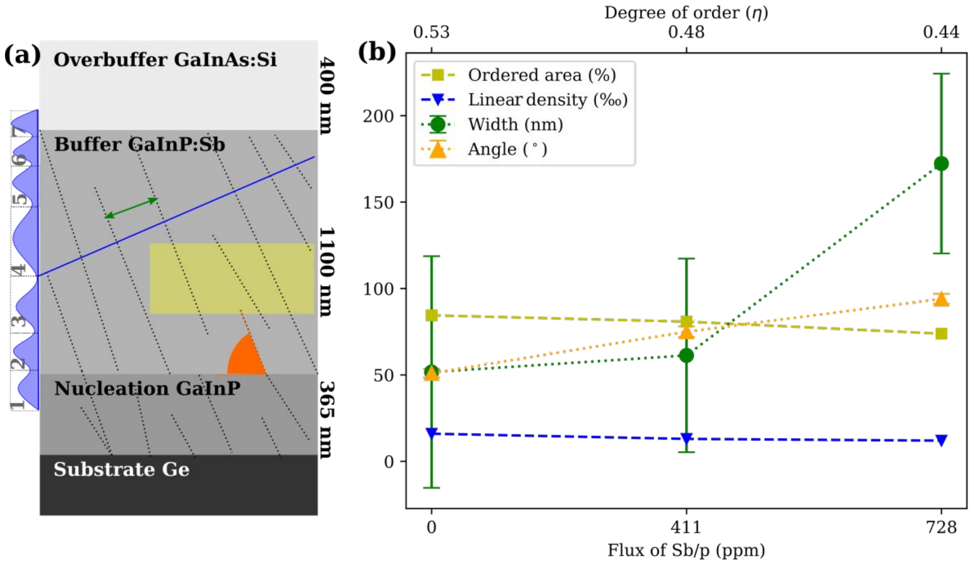

Fig. S4 (a) Scheme of the layer stack and APDBs appearance with indications to illustrate the measured parameters: domain width (green), linear density of APDBs (blue) with the intensity profile on the left side, fraction of ordered area (yellow) and angle (orange). (b) Plot of the measured parameters as a function of the degree of order / flux of Sb.

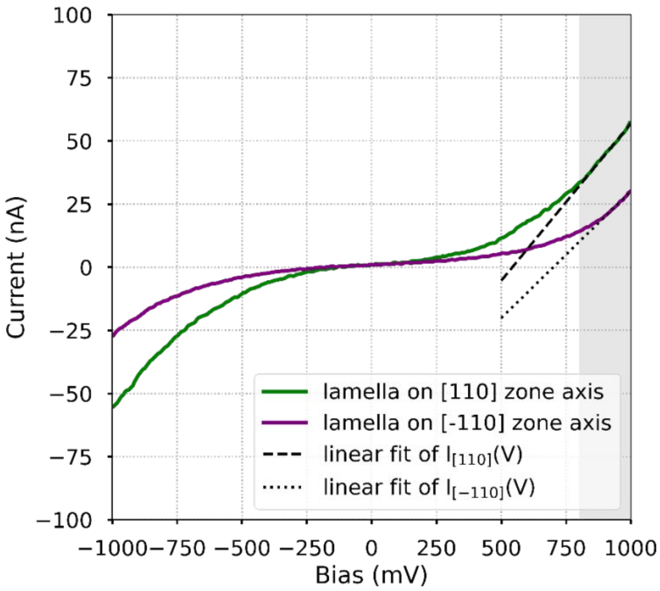

Fig. S5 Example of the I-V characteristics for M-S-M structures (tip-sample-sample support). At larger biases, the current increases almost linearly and becomes the dominating term, the resistance of the tip can be considered negligible and the I-V curve can be differentiated to obtain the resistance of the sample (in figure highlighted in grey).

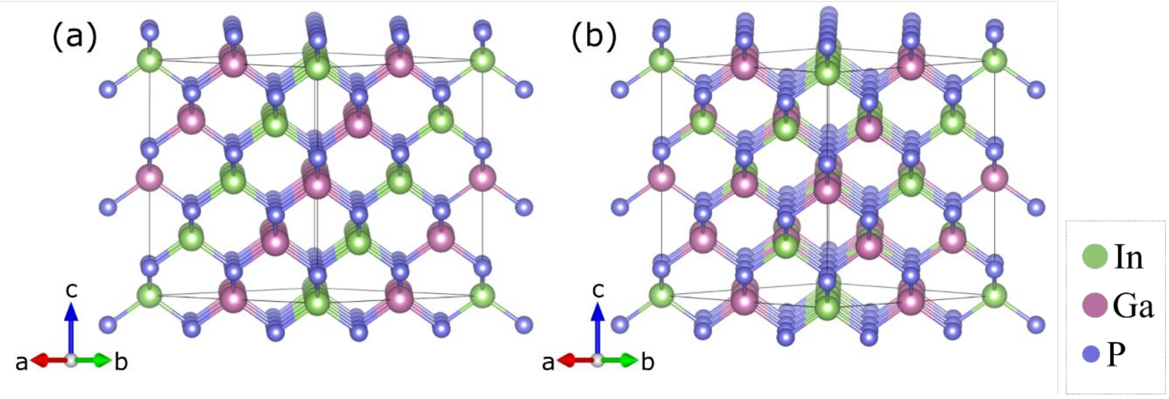

Fig. S6 Structures before relaxation:(a) Ordered GaInP. (b) Disordered GaInP.

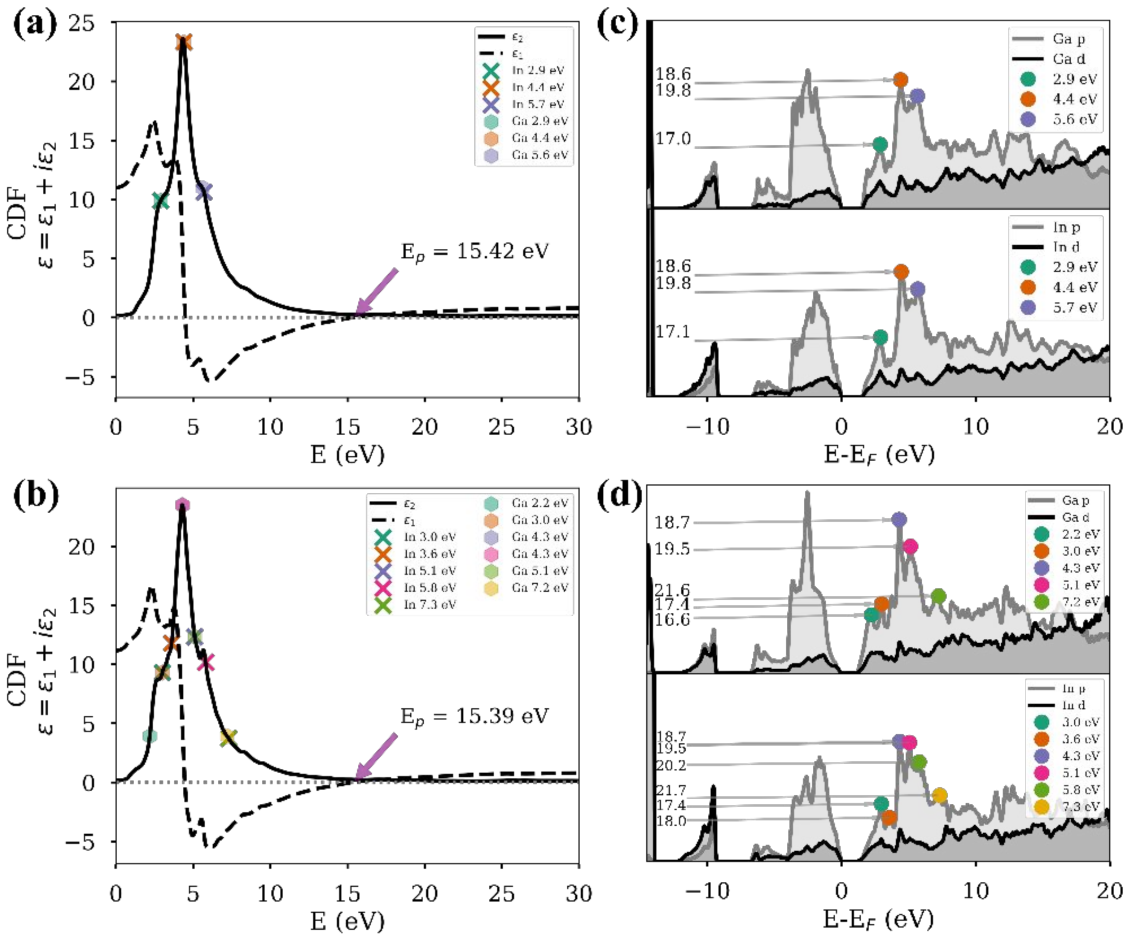

Fig. S7 Real part (solid line) and imaginary part (dashed line) of the CDF computed for the disordered (a) and ordered (b) structures. The partial density of states for the In and Ga atoms for both structures are plotted aside, (c) for the disordered structure and (d) for the ordered case. The inter-band transitions are highlighted with the same color on both plots to be uniquely identified.

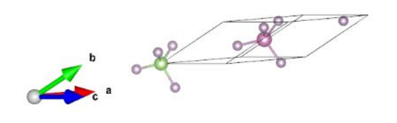


```
Lattice type          R
Space group name      R 3 m
Space group number    160
Setting number        2

Lattice parameters

    a         b         c        alpha     beta     gamma
 6.92559  6.92559  6.92559  33.4090  33.4090  33.4090

Unit-cell volume = 89.680643 Å^3

Structure parameters

                         x            y            z            Occ.      B
Site      Sym.
   1 Ga   Ga           0.00019     0.00019     0.00019     1.000     1.000
1a         3m
   2 In   In           0.49903     0.49903    -0.50097     1.000     1.000
1a         3m
   3 P    P            0.36967     0.36967    -0.63033     1.000     1.000
1a         3m
   4 P    P2           0.88111    -0.11889    -0.11889     1.000     1.000
1a         3m
=======================================================================
```

Zone axis: [1-10]

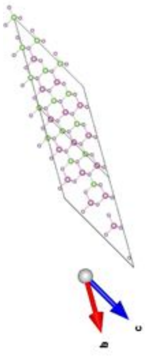

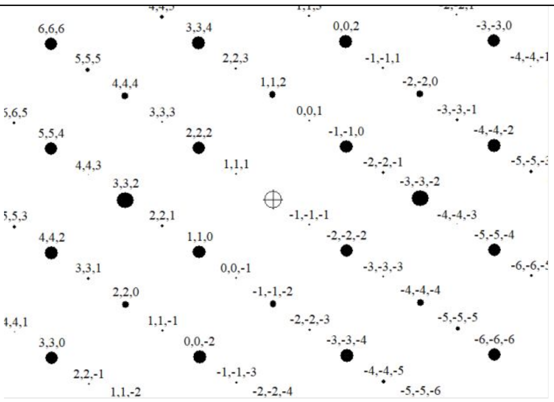

Zone axis: [11-1]

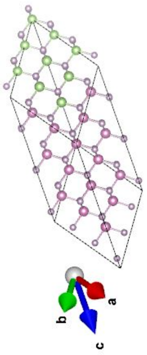

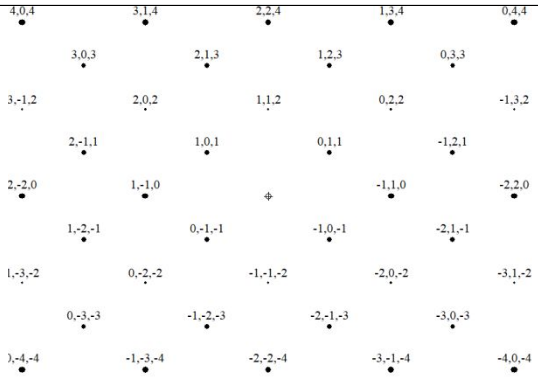

Fig. S8 Crystallographic information of GaInP R3m. A conventional 3x3x3 cell oriented along the zone axes where the ordering is visible ([1-10]) and hidden ([11-1]), are displayed together with their corresponding diffraction pattern.
